# Cost analysis of two types of fixed maxillary retainers and a removable vacuum-formed maxillary retainer: a randomized controlled trial

**DOI:** 10.1093/ejo/cjab080

**Published:** 2022-01-11

**Authors:** Mikael Sonesson, Sasan Naraghi, Lars Bondemark

**Affiliations:** 1 Department of Orthodontics, Faculty of Odontology, Malmö University, Sweden; 2 Orthodontic Clinic, Public Dental Health, Växjö, Sweden

## Abstract

**Background:**

There has been an increased interest in conducting healthcare economic evaluations. Also, orthodontic treatments have gathered focus from an economic point of view, however orthodontic research seldom examines both clinical and economic outcomes.

**Objective:**

To evaluate and compare the costs of three retention methods: a bonded retainer to the maxillary four incisors, a bonded retainer to the maxillary four incisors and canines, and a removable vacuum-formed retainer (VFR) in the maxilla. The null hypothesis was that there was no difference in costs for the three types of retention methods.

**Trial design:**

Three-arm, parallel group, single-centre, randomized controlled trial.

**Materials and methods:**

Ninety adolescent patients, 54 girls and 36 boys, treated with fixed or removable retainers in the maxilla, were recruited to the study. The patients were randomized in blocks of 30, by an independent person, to one of three groups: bonded multistranded PentaOne (Masel Orthodontics) retainer 13-23, bonded multistranded PentaOne (Masel Orthodontics) retainer 12-22, and removable VFR. A cost analysis was made regarding chair time costs based on the costs per hour for the specialist in orthodontics, and material costs plus any eventual costs for repairs of the appliance. Changes in Little’s irregularity index and in single contact point discrepancies (CPDs) were measured on digitalized three-dimensional study casts. Data were evaluated on an intention-to-treat basis. The analysis was performed at 2 years of retention.

**Results:**

No statistically significant difference in costs between the maxillary fixed retainers and the VFRs was found, however, the material and emergency costs were significantly higher for the VFR compared with the bonded retainers. All three retention methods showed equally effective retention capacity, and no statistically significant differences in irregularity or CPDs of the maxillary anterior teeth in the three groups was detected.

**Limitations:**

It was a single-centre trial, and hence less generalizable. Costs depended on local factors, and consequently, cannot be directly transferred to other settings.

**Conclusions:**

All three retention methods can be recommended when considering costs and retention capacity.

**Trial registration:**

NCT04616755.

## Introduction

Relapse is an unwanted side-effect of orthodontic treatment, both for the patient and the orthodontist (1–3). A particular concern is to keep the anterior teeth in the maxilla well aligned, as this anatomical region is of major concern for the patient (4, 5).

There are different retention strategies to maintain the position of the teeth in the maxilla. The strategies commonly include a bonded retainer between the maxillary canines (6, 7). Also removable vacuum-formed retainers (VFRs) are widely used, and VFRs have been reported to produce stability as good as that produced by fixed retainers (8). Each strategy has both advantages and disadvantages. Bonded retainers have the advantage of being independent of the compliance of the patients, but are at risk for coming unattached or being fractured (8, 9). A reduced extension of the retainer, to include only the maxillary incisors, may result in fewer complications compared with a retainer that also includes the canines. In addition, the maxillary canines seem to be more stable after treatment than the incisors, and thus, do not need to be included in the retainer (7, 10, 11). Nevertheless, the use of bonded retainers, regardless of design, makes cleaning in approximal areas more difficult, which might increase the risk for accumulation of plaque and development of gingivitis and caries (12, 13). Thus, the use of a removable retainer may facilitate the maintenance of good oral hygiene compared with the use of a bonded retainer. But a shortcoming with a removable retainer in the long run is dependency on the patient’s compliance, and thus there is a risk of relapse in cases of non-compliance (14, 15).

Recently, there has been increased interest in conducting healthcare economic evaluations. The main factor is that resources (personnel, time, facilities, and equipment) within the health sector are limited (16). While orthodontic treatments have been focussed upon from an economic point of view, it has been described and concluded that orthodontic research seldom examines both clinical and economic outcomes (17).

With resources within dental care being restricted, knowledge of effects and related costs are crucial when making treatment decisions. Thus, lack of economic analysis in dental health service may cause unsustainable over expenditure and result in decrease of services or resources in other areas of dental healthcare (18). Regarding the costs of different orthodontic retention methods, there exist only a few studies (19, 20). To the best of the authors’ knowledge, there are no studies in the literature that have explicitly evaluated and compared the costs between maxillary fixed and removable retainers during the first 2 years of retention.

Recently, we conducted a randomized controlled trial (RCT) on the stability of maxillary anterior teeth, comparing two bonded and a VFR after 2 years of retention (21). All three retention methods showed good capacity to retain the maxillary anterior teeth but 16% of the patients in the VFR group had breakage or loss of their VFRs compared with approximately 8% of the patients in each of the two groups with fixed retainers. This investigation aimed to use RCT methodology to evaluate and compare the costs of three retention methods, i.e. a bonded retainer to the maxillary four incisors, a bonded retainer to the maxillary four incisors and two canines, and a removable VFR in the maxilla. The costs of the three retention treatments were evaluated after 2 years of retention. Our null hypothesis was that there would be no differences in costs between the retention methods after 2 years of retention.

## Materials and methods

### Participants and study design

Adolescent patients treated with fixed appliance in the maxilla and mandible, or solely in the maxilla, were recruited at the Orthodontic Clinic Växjö, Public Dental Service, Region Kronoberg, Sweden. Patients with clefts or syndromes, patients with agenesis or extracted maxillary anterior teeth, and patients who underwent orthognathic surgery, were excluded. Enrolment started in October 2013 and ended in October 2017. The last follow-up of the retentions was completed in October 2019. The trial was a single-centre, RCT with three parallel arms and a 1:1:1 allocation ratio. The Regional Ethical Research Board, Linköping, which follows the Declaration of Helsinki, approved the trial (Dnr 2013/131).

### Randomization

After informed consent from each patient and their custodians was obtained, the participants were randomly allocated to one of the three retention groups as follows: 1. bonded retainer to the maxillary four incisors and two canines, 2. bonded retainer to the maxillary four incisors 12-22, and 3. removable VFR covering all maxillary teeth.

The randomization process was prepared by an independent person and carried out by three staff members not involved in the trial. The randomization used blocks of 30. Every new participant randomly picked a sealed opaque envelope and revealed their group assignment by opening the envelope. Recruitment continued until the total number of participants met the estimated sample size. Data on all participants were evaluated on an intention-to-treat (ITT) basis. Consequently, all randomized patients remained in the allocated group and were followed during the 2 years of retention.

Before the retention period, the patients were treated with a pre-adjusted fixed appliance in the maxilla, or in the maxilla and the mandible (0.022 slot size, MBT prescription, Victory Series, 3M Unitek, Monrovia, California, USA). The treatments were ended when the treatment goal was achieved, thus the overjet, overbite, and occlusion had to be normalized before the retention period was started. Records of the patients included study casts produced before orthodontic treatment (T0), after removal of the fixed appliance (T1), and after 2 years of retention (T2). The retention check-ups at the orthodontic clinic were performed at three scheduled occasions: 1 month, 1 year, and 2 years after insertion of the retainers. All three types of retainers were manufactured by dental technicians outside the clinic.

### The fixed retainers

**Figure 1.**
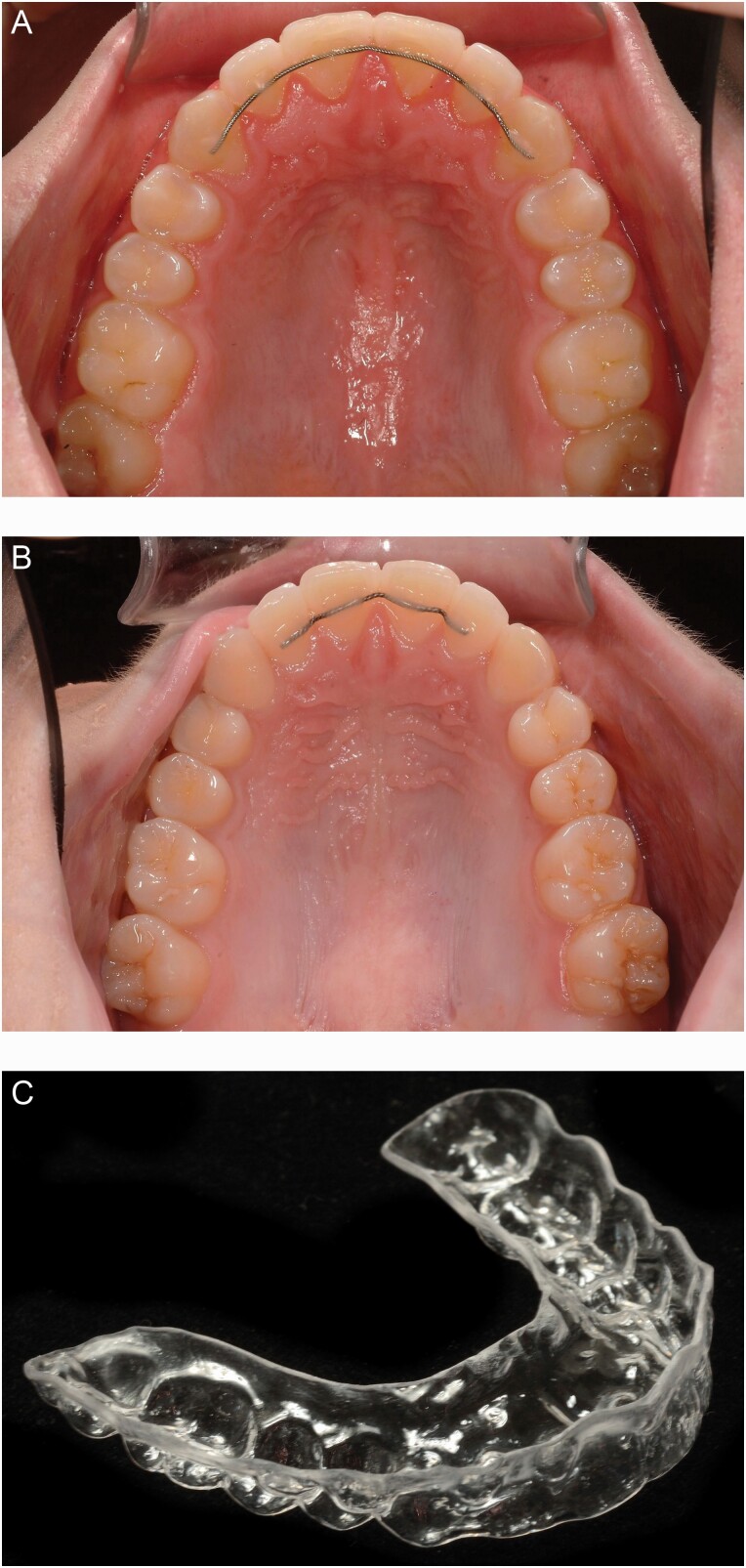
(A) Bonded retainer 13-23, (B) bonded retainer 12-22, and (C) vacuum-formed retainer 17-27.

The retainers in group A (maxillary four incisors and two canines 13-23, Figure 1A) and group B (maxillary four incisors 12-22, Figure 1B) were manufactured from PentaOne 0.0195, Masel, Carlsbad, California, USA. These retainers were bonded with Transbond Supreme LV (TSLV-3M, Unitek, Monrovia, California) to the lingual surface of each tooth and passed the level of the contact points of the teeth from right canine to left canine (group A) and from right lateral incisor to left lateral incisor (group B). The retainers were bonded by three experienced staff members after removal of the fixed appliances.

### The vacuum-formed retainer

The VFRs in group C (Essix™, Erkodur, 1.5 mm 120 ø, Erkodent® Erich Kopp GmbH, Pfalzgrafenweiler, Germany) covered all maxillary teeth (Figure 1C). The VFRs were produced within 1 day after debonding. The patients followed a standard protocol for VFR-wear: 22–24 hours/day during the first 4 weeks, then every night. After 1 year of retention, the wearing time was reduced to every other night.

### Outcome measures

The outcome measures were: 1. Total mean costs as chair time costs based on the costs per hour for the specialist in orthodontics; 2. material costs and any additional costs for repairs of the appliance. The post-treatment changes in irregularity of the six maxillary anterior teeth, according to Little’s irregularity index (LII) (22) and contact point discrepancies (CPDs) of the maxillary anterior teeth, were registered as presented elsewhere (21).

### Cost analysis

With results and consequences of each group’s retentions presumably being equivalent to each other, i.e. all three groups had retention during 2 years with the intention to stabilize the corrected maxillary anterior teeth, the difference between the groups was reduced to a comparison of costs.

Orthodontic staff registered the chair time and whether the appointment was planned or an emergency visit. The treatment time costs and emergency costs including staff, room, room maintenance, dental equipment, and sanitation were based on the costs per hour for a specialist dentist (3400 SEK/€333) according to the price list of 2019 for specialist dentistry in the Public Dental Service in Region Kronoberg, Sweden. Based on the prices for the year 2019, the material costs were derived by the dental technician costs (fixed retainer; 485 SEK/€48.0, Essix; 520 SEK/€51.5) plus any eventual costs for repairs of the appliance. All costs were expressed in Euros (€), SEK 100 = €9.9 at a mean currency value (www.xe.com). The dental care including orthodontic treatment was funded by the publicly healthcare system, with a per capita compensation irrespective of type of retainer treatment.

### Irregularity index and CPDs

When evaluating post-treatment stability, i.e. the changes in LII, these changes were assessed between start of retention (T1) and at the 2-year follow-up (T2). In addition, maximum CPD was assessed as the most severe single contact point per participant.

The measurements were made manually on digitalized three-dimensional (3D) study casts for the three retention groups before treatment with fixed appliance (T0), start of retention (T1), and at the 2-year follow-up (T2). The study casts were digitized with a stationary 3D scanner (D3, 3Shape, Copenhagen, Denmark) prior to the measurements. On the digital models, the measurement points were located using the OnyxCeph^3^™ software (v3.2.142, Image Instruments, Chemnitz, Germany) with semi-automatic segmentation (21).

### Intention-to-treat

Data on all participants were evaluated on an ITT basis. Consequently, all randomized patients remained in the allocated group. Subsequently, patients with discontinued observation or lost to follow-up were still included in the final analysis by assessing the group’s maximum value for costs and cost changes, considering the primary outcomes (retention treatment costs, material costs, emergency costs) as well as for the secondary outcome variables representing the change in irregularity index and CPDs.

### Sample size calculation

A sample size calculation was made with the intention of detecting a clinically relevant and realistic difference in cost of 500 SEK/€49.3 (SD 500/49.3) between the three retention groups. Consequently, using a power of 90% and *α* = 0.05, the sample size of each group was estimated to be 28. The sample size calculation for comparison of the retention capacity of the three retention methods has been published elsewhere (21).

### Statistical analysis

Data on costs were processed with the IBM-SPSS software (version 27.0, Chicago, Illinois USA). Analysis of variance with Tukey’s *post hoc* test was used to compare the costs within and between the retention groups. Differences in LII and CPD between the three retention groups were tested as specifically described elsewhere (21). Continuous variables were tested by Kruskal–Wallis and categorical variables by chi-square test using the programming language R (v. 4.0.2) (23). Differences with probabilities of less than 5% (*P* < 0.05) were considered statistically significant.

## Results

**Figure 2.**
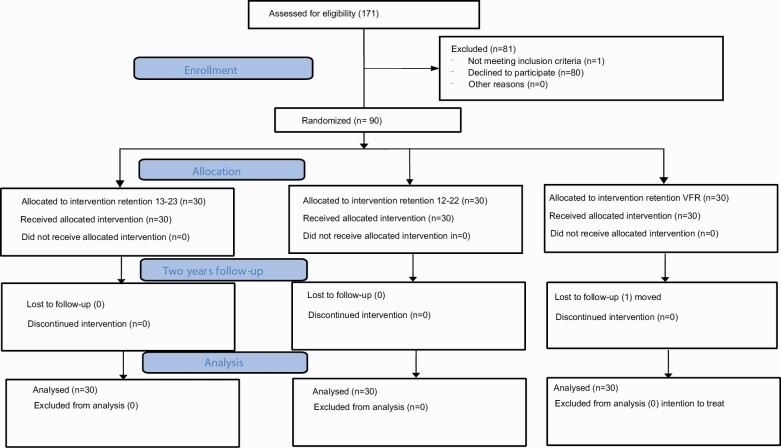
Consort flow chart.

Ninety patients, 54 females and 36 males, with a mean age of 15.9 years at start of the retention period, were recruited and the participants were followed for 2 years. All patients remained within the groups during the trial except one participant from group C (VFR) who changed domicile but, according to the ITT principle, was still included in the cost and retention capacity analysis Table 1; (Figure 2). In group A (13-23), 9 patients had 16 emergency visits due to composite breakage and 1 patient lost the retainer twice. In group B (12-22), five patients had seven emergency visits due to composite breakage and one patient lost the retainer. In group C (VFR), 16 patients had 19 emergency visits due to breakage or loss of the retainer.

**Table 1.** Demographic data at retention start, duration of treatment with fixed orthodontic appliance and duration of retention treatment.

Group	Gender	n	Age, years, mean (SD)	Treatment, months, mean (SD)	Retention, months, mean (SD)
Bonded retainer 13-23	Female	17	15.7 (1.8)	24.7 (9.6)	24.6 (2.4)
	Male	13	16.0 (1.4)	23.7 (11.8)	24.9 (1.4)
	Total	30	15.8 (1.6)	24.3 (10.4)	24.7 (2.0)
Bonded retainer 12-22	Female	20	15.7 (1.8)	21.4 (9.8)	25.5 (1.5)
	Male	10	16.4 (2.2)	24.2 (3.8)	24.7 (1.6)
	Total	30	15.9 (1.9)	22.3 (8.3)	25.2 (1.5)
Vacuum-formed retainer	Female	17	15.5 (2.1)	19.8 (7.5)	25.3 (1.9)
	Male	13	16.6 (1.8)	30.8 (16.0)	25.5 (1.5)
	Total	30	16.0 (2.0)	24.6 (12.9)	25.4 (1.7)
*P* value three-group comparison		0.659	0.893	0.852	0.288
Total population	Female	54	15.6 (1.9)	21.9 (9.1)	25.2 (1.9)
	Male	36	16.3 (1.8)	26.4 (12.2)	25.0 (1.4)
	Total	90	15.9 (1.9)	23.7 (10.6)	25.1 (1.7)

*P* values calculated with Kruskal–Wallis for numerical variables and chi-square test for categorical variables.

### Cost analysis

#### Total retention costs

The mean total retention cost per patient was €734 for the bonded retainer to the maxillary four incisors and two canines (13-23), €674 for the bonded retainer to the maxillary four incisors (12-22), and €778 for the VFR. No statistically significant difference in total cost was seen between the three retention methods (Table 2).

**Table 2.** Treatment time, material, emergency, and total costs (Euro) for retention with bonded retainer 13-23, bonded retainer 12-22, and maxillary vacuum-formed retainer. Mean, SD (standard deviation), CI (confidence interval), and group difference are presented.

	Bonded retainer 13-23 (*n* = 30)			Bonded retainer 12-22 (*n* = 30)			Vacuum-formed retainer (*n* = 30)			Difference between groups* (*P*)
	Mean	SD	95% CI	Mean	SD	95% CI	Mean	SD	95% CI	
Treatment time costs	633	127	586 to 680	600	74	572 to 627	579	138	527 to 630	NS
Material costs	51	17	44 to 57	49	9	46 to 52	83	37	70 to 97	<0.0001**
Emergency costs	50	127	3 to 97	25	70	−1 to 51	116	121	71 to 161	0.005***
Total costs	734	270	633 to 834	674	149	618 to 729	778	291	669 to 887	NS

No retreatment was needed in any group.

*One-way analysis of variance with Tukey’s *post hoc* test.

^**^Material costs were statistically significant lower for bonded retainers between 12-22 and 13-23 compared with vacuum-formed retainer.

^***^Emergency costs were statistically significant lower for bonded retainer between 12-22 compared with vacuum-formed retainer.

#### Treatment time costs

The mean cost for treatment time per patient was €633 for the bonded retainer to the maxillary four incisors and two canines (13-23), €600 for bonded retainer to the maxillary four incisors (12-22), and €579 for the VFR. No statistically significant difference between the retainers was found (Table 2).

#### Material costs

The average material cost was €51 for the bonded retainer to the maxillary four incisors and two canines (13-23), €49 for bonded retainer to the maxillary four incisors (12-22), and €83 for the VFR. The material cost was significantly higher for the VFR compared with the two bonded retainers (*P* < 0.0001) (Table 2).

#### Emergency costs

The mean emergency cost per patient was €50 for the bonded retainer to the maxillary four incisors and two canines (13-23), €25 for bonded retainer to the maxillary four incisors (12-22), and €116 for the VFR. The emergency cost was significantly higher for the VFR than for the two bonded retainers (*P* = 0.005) (Table 2).

No statistically significant group difference was found in costs regarding absence from appointments. For the bonded retainer to the maxillary four incisors and two canines (13-23) as well as for the VFR, the cost per patient was €27 while the cost for the bonded retainer to the maxillary four incisors (12-22) was €11.

### Irregularity index and CPDs

All three retention methods showed good capacity to retain the maxillary anterior teeth. No statistically significant differences in irregularity of the maxillary anterior teeth in the three groups were detected. These data are specifically presented elsewhere (21).

## Discussion

### Main findings

This trial was the first healthcare economic evaluation to assess costs of fixed and removable maxillary retainers and no difference in costs or retention capacities were found between the retention methods. Although, the material and emergency costs were significantly higher for the VFR compare to the bonded retainers, no statistically significant difference for the total retention costs was seen between the fixed retainers and the VFR, or between the two types of fixed retainers. Thus, the results of this trial confirm our hypothesis that there are no differences in costs between the three retention methods. Also, the three methods, without any difference between them, showed good clinical capacity to retain the maxillary anterior teeth; therefore, all three retention methods can be recommended both regarding retention capacity and costs.

All the three types of retainers were manufactured by dental technicians outside the orthodontic clinic. It is conceivable that the material costs can be reduced if the retainers are manufactured at the clinic by a dental nurse or a dentist or even by a 3D printer. It was shown in the present trial that, even if the material and emergency costs were higher for the VFR, due to repeated damages or loss of the retainers, the total retention costs did not differ significantly between the three retainers. This might be explained by a shorter initial chair time at the clinic for the patients with VFRs compared with the patients with fixed retainers. Accordingly, the slightly higher treatment costs for the bonded retainers compared with the VFR equalizes the higher material cost for the VFR. The differences between the total costs of the different retention methods are not therefore significant.

It can be pointed out that new techniques for removable retainers may impact costs and improve the ability to predict the outcome of the retention. Patient compliance can be improved by artificial intelligence or by devices such as thermo-sensitive microsensors. Their clinical application may reduce retention costs in the future (24, 25).

### Strengths and generalizability

This study was implemented alongside an RCT regarding the stability of maxillary anterior teeth with fixed or removable retainers (21). The randomization process was performed by a person not involved in the trial or treatment. It can also be pointed out that the randomization *per se* implies that selection bias is avoided, and most importantly, any confounders were evenly distributed between the groups. In addition, the results were evaluated on an ITT basis and the attrition bias was low since there was only one drop-out in the trial.

The patients who were included in the trial were diverse in terms of gender distribution, age, and treatment at a specialist clinic, and thus, were considered representative of orthodontic patients. Hence, the design and performance of this trial allowed the results to be implemented in daily orthodontic practice.

### Limitations

This trial was conducted at one orthodontic clinic which may involve a higher risk of bias compared with multi-centre studies due to a limited trial population, few operators, small number of involved staff members, and commendable organization (26).

The number of patients who initially were not interested in being enrolled and participating in the trial was rather high, which therefore increased the risk of selection bias. The main reason for not enrolling in the trial was that these patients looked forward to having their treatment completed and not being further evaluated in a research trial.

It is important to point out that monetary variables are influenced by local factors such as insurance systems, staff salaries, rental costs, taxes, urban versus rural areas, etc., and consequently, the figures shown in this trial cannot be directly extrapolated to other locations. Thus, the generalizability of the findings to other settings may be limited as the study was carried out on a regional scale.

Another limitation is that the costs consisted of so-called direct costs. Hence, the trial did not include indirect costs, i.e. costs defined as loss of income (wages plus social security costs) incurred by the patients’ parents’ absence from work to accompany the patient to the orthodontic appointment, including waiting time at the clinic and the travelling duration. However, the direct costs normally comprise the majority (65–90%) of the total costs (27, 28) and it may be assumed that probably the indirect costs would not differ between the different retention groups in this trial. Still, we do not have a complete picture of the total costs. Nevertheless, the direct costs of the retention treatment, that constitutes a significant part of the costs of the overall orthodontic treatment, is important to include when costs of the entire orthodontic treatment is performed.

## Conclusions

The use of maxillary fixed and removable VFRs showed no significant differences in costs, as well as good clinical capacity to retain the maxillary anterior teeth. Thus, all three retention methods can be recommended when considering costs and retention capacity.

## Data Availability

The data underlying this article were provided by Region Kronoberg, Sweden under licence/by permission. Data will be shared on request to the corresponding author with permission by Region Kronoberg, Sweden.

## Trial registration

www.clinicaltrials.com (NCT04616755).
